# Surgical resection of a massive residual retroperitoneal mass after chemotherapy for a paratesticular rhabdomyosarcoma: a case report

**DOI:** 10.1186/s13256-022-03707-x

**Published:** 2022-12-23

**Authors:** Takoua Chalouati, Montassar Ghalleb, Amani Jallali, Maher Slimane, Ghada Sahraoui, Jamal Ben hassouna, Riadh Chargui, Khaled Rahal

**Affiliations:** 1 Surgical Oncology Department, Salah Azaiez Institute of Oncology, Tunis, Tunisia; 2 Histology Department, Salah Azaiez Institute of Oncology, Tunis, Tunisia; 3 Faculty of Medicine of Tunis, University Tunis El Manar, Tunis, Tunisia

**Keywords:** Paratesticular rhabdomyosarcoma, Paraaortic lymph nodes, Surgery

## Abstract

**Introduction:**

Paratesticular rhabdomyosarcoma is a rare and aggressive mesenchymal tumor, accounting for only 7% of all rhabdomyosarcomas. It is mainly encountered in children and adolescents. The standard treatment consists of radical orchidectomy with negative surgical margins. However, chemotherapy is recommended to control retroperitoneal micrometastasis. The place of surgery for progressive retroperitoneal lymph node metastases remains controversial. We present a case of paratesticular rhabdomyosarcoma with progressive retroperitoneal lymph node metastases treated with surgery.

**Case report:**

We report a case of a 17-year-old North African male with no particular medical history who presented with a left scrotal mass that had been evolving for several months. Beta-human chorionic gonadotropin, alpha-fetoprotein, and lactate dehydrogenase were normal. Scrotal ultrasonography revealed the presence of a 6 cm heterogeneous hypoechogenic tissular mass with cystic areas adherent to the left scrotal wall, which was thickened in some places and vascularized by color Doppler. It exerted a mass effect on the homolateral testicle, which was of average volume. The thoracic–abdominal–pelvic computed tomography scan showed the presence of suspicious paraaortic lymph nodes. The most voluminous one measured 16 × 23 mm^2^. A left orchidectomy was performed. The final pathology report revealed an 8 cm paratesticular rhabdomyosarcoma of the embryonic type that displaced the testicle without invading it. Without going beyond it, it infiltrated the epididymis, the rete testis, and the albuginea. The surgical margin at the level of the spermatic cord was free. The patient had adjuvant chemotherapy (ifosfamide, vincristine, and dactinomycin). The patient had a challenging paraaortic lymph node dissection since the mass enlaced the left ureter and renal vessels. On histological examination, the paraaortic lymph nodes were metastatic.

**Conclusion:**

Rhabdomyosarcoma is an aggressive malignancy with high metastatic potential. Therefore, only an accurate diagnosis and early treatment can ensure better survival. Surgery in expert hands seems to be a good option for progressive retroperitoneal nodes. However, further studies are needed to determine the place of surgery in this setting.

## Introduction

Paratesticular rhabdomyosarcoma (RMS) is a rare and aggressive tumor [1], accounting for only 7% of all RMS [2]. It is mainly found in children and adolescents. Embryonic and alveolar variants are the two most common subtypes [3].

The primary differential diagnoses are scrotal emergencies that must be excluded [4, 5].

Owing to the rarity of the disease, the management strategy is based on the management of non-seminomatous testicular tumors [3]. It is a multimodal treatment approach, often using surgery, radiation, and chemotherapy [6]. What to do when faced with borderline resectable paraaortic masses remains controversial.

We aim to report a surgically challenging metastatic paratesticular RMS case and shed more light on this rare disease.

## Case report

We report the case of a 17-year-old North African male with no particular medical or family history, studying at high school, who presented with a left scrotal mass that had been evolving for several months. The patient was not given any special medication prior to diagnosis. The patient has no history of smoking or alcohol consumption.

On physical examination, there was an 8 cm painless left scrotal tumor. The light transmittance test was negative. The patient did not show any sign of testosterone deficiency, that is, testosterone levels were not measured. No regional lymph node areas were clinically palpable. The neurological examination was normal and no significant clinical findings were detected on other physical examinations.

The tumor markers [alpha-fetoprotein, beta-hCG, and lactate dehydrogenase (LDH)] were within normal ranges.

The ultrasound (US) revealed the presence of a 6 cm heterogeneous hypoechogenic tissular mass with cystic areas adherent to the left scrotal wall, which was thickened in some places and vascularized by color Doppler. It exerted a mass effect on the homolateral testicle, which was of average volume with a loss of contours in areas more marked at the level of the inferior pole. The thoracic–abdominal–pelvic computed tomography (CT) scan showed some suspicious lateral–aortic enlarged lymph nodes. The largest of these was 16 × 23 mm.

One week later, the patient had a left inguinal orchidectomy after first and high ligature of the spermatic cord with full resection margins.

The final pathology report concluded with an 8 cm paratesticular rhabdomyosarcoma of embryonic type that displaced the testicle without invading it. Without going beyond it, it infiltrated the epididymis, the rete testis, and the albuginea. The surgical margin at the level of the spermatic cord was free.

The patient was staged as stage III and was planned for adjuvant polychemotherapy implemented in nine cycles, combining these molecules: ifosfamide, vincristine, and actinomycin D. Chemotherapy was started 1 month later. He only received six cycles and refused further chemotherapy.

A CT scan after six chemotherapy cycles showed a residual paraaortic 5 cm mass, with no other sign of metastatic disease.The patient had a positron emission tomography (PET) CT that showed a 4 cm hypermetabolic mass at the level of the renal pelvis; no other hypermetabolic location was present.

After discussion with the patient and his guardian, the multidisciplinary meeting decided to perform a paraaortic lymph node dissection. The patient expressed an apparent refusal of any systemic treatment and wanted to have the disease surgically removed. Radiation therapy was also discussed, but it was ruled out due to the absence of an immediately available appointment.

The surgery was performed 4 months after the second CT scan and 2 months after the PET CT. On admission, the examination was strictly normal. The patient was afebrile and had normal vital signs; pulse rate was 84 beats per minute and blood pressure was within normal measures of 120/60 mmHg. The patient did not receive any particular treatment, except for prophylactic anticoagulation one injection of enoxaparine 0, 6 UI per day.

All the laboratory findings were normal (Table 1).

**Table 1:** Results of biological blood analyses

Blood analyses	Results/unit
Hemoglobin	14.3 g/dl
Leukocytes	6780 cells/mcl
Platelets	375,100 cells/mcl
Urea	5.6 mmol/L
Creatinine	64 μmol/L
C-reactive protein (CRP)	3 mg/L
Potassium	4.1 mmol/L
Sodium	132 mmol/L
Aspartate transferase (AST)	15 IU/L
Alanine transaminase (ALT)	16 IU/L
Total bilirubin (T-bil)	10 μmol/L
LDH	115 U/L
Beta-hCG	5.6 IU/L
Alpha-fetoprotein	6 ng/ml

CRP: c-reactive protein, AST: aspartate transferase, ALT: alanine transaminase, T-bil: Total bilirubin, LDH: lactate dehydrogenase, Beta-hCG: beta chorioglobulin hormon

We started with laparoscopy, which found a 2 cm relapse at the level of the section of the left spermatic cord. The extraperitoneal approach was initially considered, but the mass was deemed unresectable with laparoscopy. We converted to midline laparotomy and first resected the spermatic cord relapse.

**Fig. 1:**
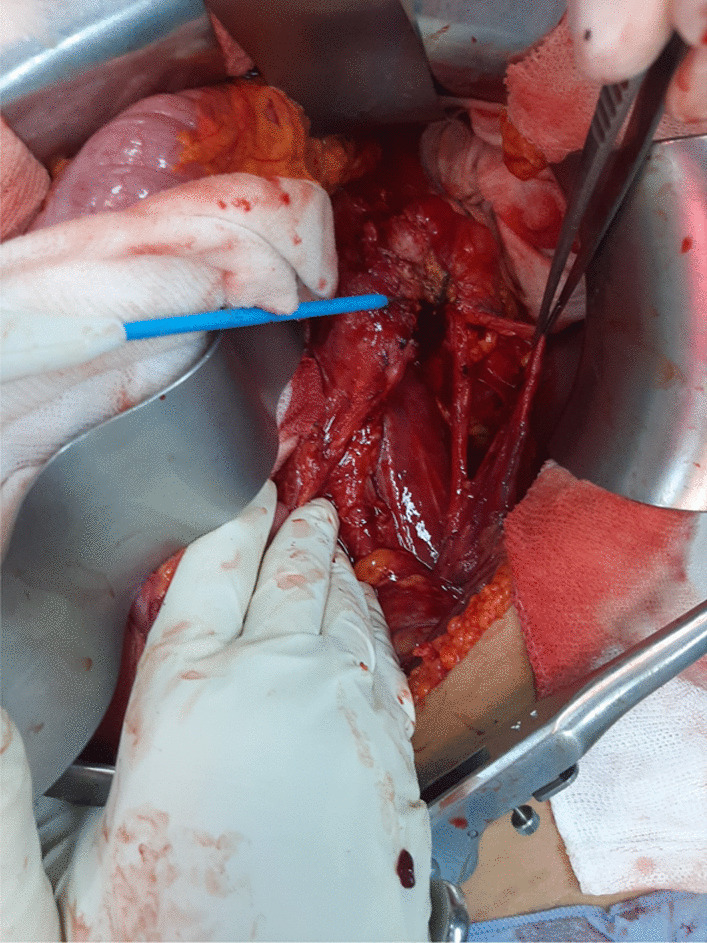
Per operative view showing the residual mass enlacing the left ureter and renal vessels

**Fig. 2:**
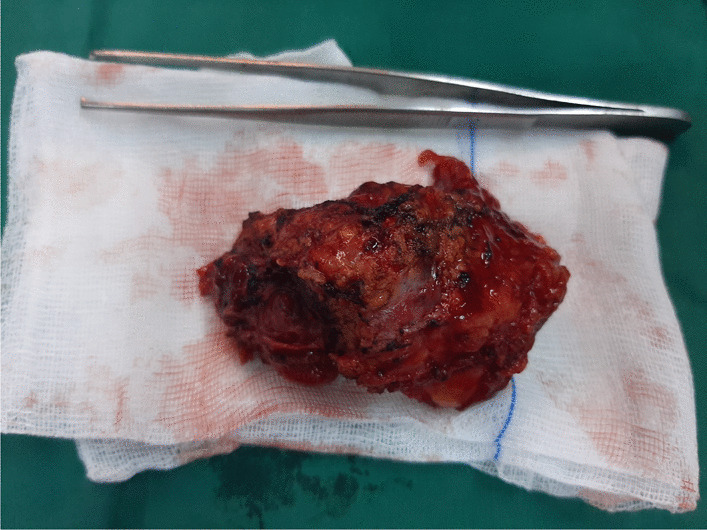
Macroscopic view of the resected specimen

**Fig. 3:**
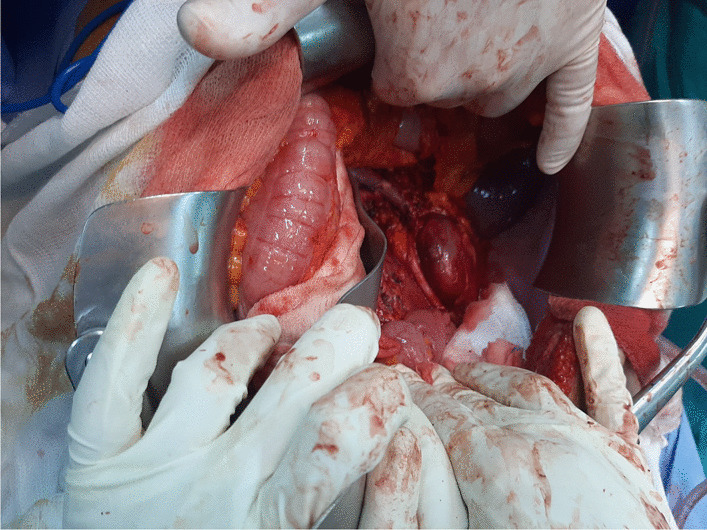
Per operative view showing the left retroperitoneal space freed of tumor and the left kidney preservation

The residual mass was 8 × 4 cm and enlaced the left ureter and the left renal vessels at the level of the renal pelvis (Fig. 1). Careful dissection helped free the ureter and the renal vessels without harming them, allowing for renal conservation (Figs. 2 and 3).

The surgery was performed by a skilled professor and lasted 180 min. There was no significant blood loss. At the end of the surgery, there was no macroscopic disease left.

**Fig. 4:**
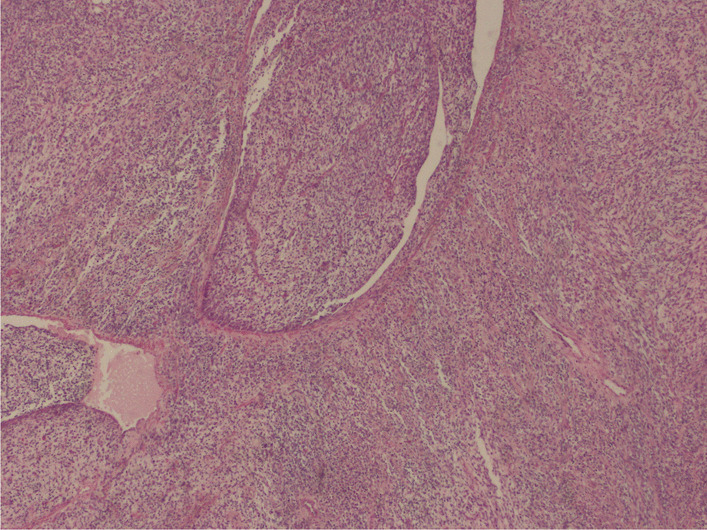
Tumor is cellular, composed of sheets of small, stellate, spindled, or round cells

**Fig. 5:**
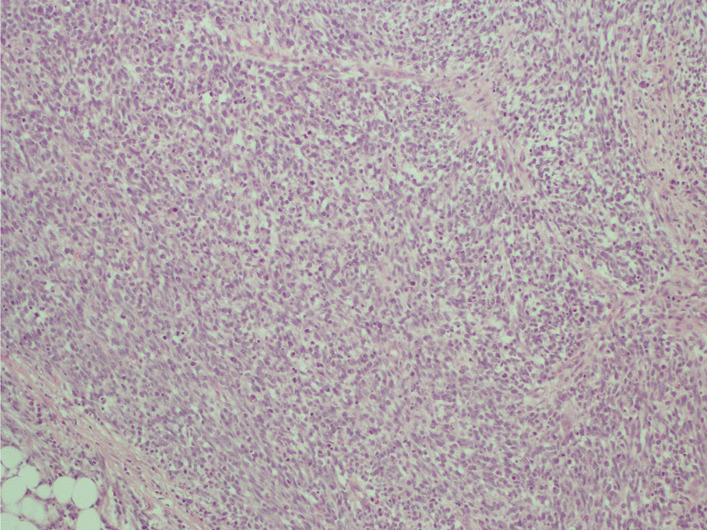
Tumor containing both hyper- and hypocellular areas with a loose, myxoid stroma

**Fig. 6:**
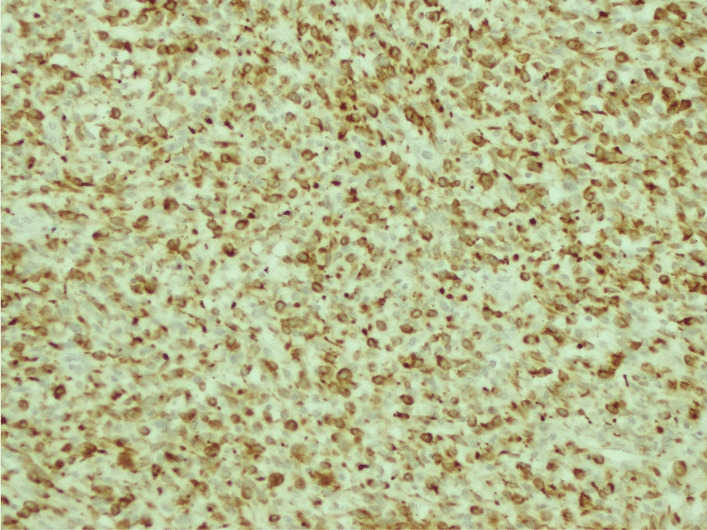
Desmin cytoplasmic positivity of tumor cells

**Fig. 7:**
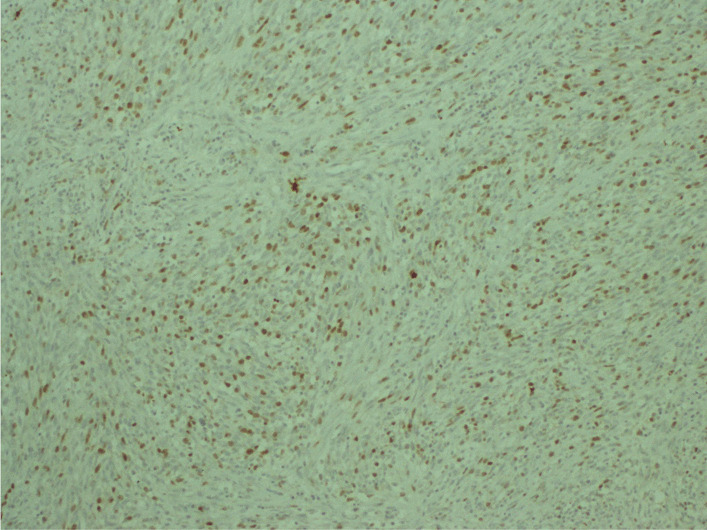
Myogenin focal nuclear staining of tumor cells

The histological examination found a cellular tumor composed of sheets of small, stellate, spindled, or round cells (Fig. 4). The tumor contained hyper- and hypocellular areas with a loose myxoid stroma (Fig. 5). Small, stellate, spindled, round cells with scant or deeply eosinophilic cytoplasm and eccentric, small oval nuclei with a light chromatin pattern and inconspicuous nucleoli. Figures of mitosis were numerous. On immunohistochemistry examination, the tumor showed cytoplasmic positivity for desmin and focal nuclear staining for myogenic (Figs. 6 and 7). The final histology examination resulted in metastatic lymph node from RMS.

The multidisciplinary meeting decided to complete two cycles of chemotherapy and perform paraaortic external beam radiation. After 7 months of surveillance, the patient completed chemotherapy and radiation. He was last seen at our department 1 year after surgery and was still disease free. The planned follow-up for this patient will be as follows: physical examination every 3 months in the first 2 years with a thoracic–abdominal–pelvic CT scan every 6 months; carried out at the initial hospital.

## Discussion

We reported the case of 17-year-old male patient with paratesticular rhabdomyosarcoma with progressive paraaortic lymphnodes, treated with debulking paraaortic surgery. Despite being difficult, the surgery was performed by an expert surgeon. Careful dissection also helped save the patent’s kidney. One year after surgery, the patient was last seen with no sign of relapse.

Rhabdomyosarcoma (RMS) is the most common soft tissue sarcoma in children. It accounts for 5–10% of all malignant tumors. It arises from primordial mesenchymal cells with different levels of skeletal muscle differentiation [7].

Paratesticular localization represents only 7% of all RMS [2]. It may arise from the spermatic cord, epididymis, or testicular tunics [7].

The age distribution is bimodal, with peaks in childhood (1–5 years) and adolescence (16–18 years) [6].

The symptoms of paratesticular RMS are non-specific [2]. It often manifests as a painless scrotal mass but can also present as bruising, hydrocele, or hernia [6].

Scrotal emergencies (testicular torsion, epididymal–orchitis, mumps orchitis) are the primary differential diagnoses and must be excluded [2, 6].

B-HCG and alpha-fetoprotein (AFP) tumor markers are usually not elevated in the case of paratesticular RMS [6].

Testicular ultrasonography is the first line preferred imaging technique [5, 6], and it is required to distinguish between testicular and paratesticular lesions [6]. In 80% of cases, it reveals a mass of heterogenous echostructure with inguinoscrotal extension [1] and increased vascularity on color Doppler [2].

Para testicular RMS can spread through the blood or the lymphatic system. The most common metastatic sites are the lung, liver, and bone. The paraaortic lymph nodes are reportedly involved in 26–43% of cases [5].

A thoracic–abdominal–pelvic CT scan is necessary as part of the extension assessment.

It is essential to get cross-sectional imaging of the retroperitoneum at the start of the process to look for patients with enlarged retroperitoneal lymph nodes (RPLN) [8]. The most common histological subtypes of RMS are alveolar and embryonal. Pathologically, the embryonic variant is characterized by poorly differentiated cells and rhabdomyoblasts with abundant eosinophilic cytoplasm[2].

The rhabdomyoblast is the characteristic cell for diagnosis. When rhabdomyoblasts are absent, immunohistochemical studies with a panel of antibodies containing myosin and desmin are used [9].

Loss of heterozygosity on chromosome 11’s short arm is a cytogenetic characteristic of this malignancy [2].

The management strategy is well codified and depends on the tumor stage and the prognostic group [3].

For tumors in the paratesticular or spermatic cord area, a radical orchidectomy should be done through an inguinal approach. Tumor resection should not be done through the scrotum. *En bloc* resection should be done for tumors invading scrotal skin. Testicle-preserving approaches should be avoided [8].

All patients, regardless of age, underwent an RPLN assessment if diagnosed with paratesticular rhabdomyosarcoma [10].

Regardless of imaging results, all patients over 10 years of age should have an ipsilateral infrarenal nerve-sparing surgical RPLN assessment. Patients who are under 10 years of age and have not had any radiographic nodal enlargement should not have a surgical RPLN assessment [8]. Occult metastases can be eradicated with multidrug therapy. Therefore, it is recommended for all prognosis groups with a significant improvement in overall survival (OS) and progression-free survival [11]. In the case of residual paraaortic masses after chemotherapy and radiation therapy and the case of negative PET CT, most authors do not recommend surgery due to its high morbidity [10]. However, in the case of positive PET CT, surgery in expert centers should be considered a viable option [12]. Hamilton *et al*. [12], in their analysis of the SEER database, found a statistically significant improvement in the 5-year overall survival of adolescent patients treated with RPLN (92%) as compared with those who did not undergo RPLN (64% *P* = 0.003).However, RPLN did not improve 5-year OS among children (98% versus 94%; *P* = 0.42) or adults (70% versus 53%, *P* = 0.64). Rhee *et al*. [13], in the update on pediatric rhabdomyosarcoma from the APSA cancer committee, stated that aggressive surgical resection may be indicated for local or regional recurrence, with complete resection improving overall survival from 8% to 37%. In our case, surgery was decided on the basis of the impossibility of performing an entire course of treatment (chemotherapy and radiation), the positive PET CT, and the growth of the residual mass. The main difficulty in our case was the intimate contact between the paraaortic mass, the left ureter, and the renal vessels. With the presence of a trained surgeon, good knowledge of anatomy, and careful dissection, the complete resection of the mass was performed without harming the left kidney. This case also highlights the importance of treating these patients in an expert center.

In the treatment of rhabdomyosarcoma, several chemotherapy regimens have been employed. The most widely used combinations are ifosfamide, vincristine, etoposide (IVE), ifosfamide, vincristine, dactinomycin (IVA) or vincristine, dactinomycin, cyclophosphamide (VAC) [11]. Radiotherapy is an essential therapeutic tool in treating rhabdomyosarcoma because it improves local control [11].

## Conclusions

Paratesticular rhabdomyosarcoma is a rare and aggressive condition. Localized forms have a good prognosis, whereas metastatic forms show inferior results. Only an accurate diagnosis and early treatment will offer higher chances of survival. Despite being challenging, surgery seemed a good alternative for progressive retroperitoneal lymph nodes in this case.

## Data Availability

Data supporting our findings were taken from the patient’s folder.
